# Visual acuity outcome in patients with diabetic maculopathy

**Published:** 2014

**Authors:** R Serban, M Cioboata, C Chiotan, C Cornăcel, R Liora, A Anghelie

**Affiliations:** * Ophthalmology Emergency Hospital Bucharest; ** Department of HIV / AIDS, Office of Ophthalmology, ”Prof. Dr. Matei Bals” Institute of Infectious Diseases, Bucharest

**Keywords:** Diabetic Maculopathy, clinically significant macular edema, Triamcinolone acetonide, bevacizumab, Visual acuity

## Abstract

The purpose of this study is to follow up visual acuity in patients diagnosed with clinically significant macular edema and treated by an intravitreal injection of triamcinolone acetonide or in combination with bevacizumab. The working method: based on the selectivity criteria we involved 295 patients (460 eyes), divided into 2 groups according to the treatment administered and one control group.

The results showed a better preservation of the functional parameter for the group of patients treated with intravitreal injection of triamcinolone acetonide and bevacizumab.

**Abbreviations:** VA = visual acuity, VA0 = visual acuity at study entry, WESDR = Wisconsin epidemiological study of diabetic retinopathy, ETDRS = Early treatment diabetic retinopathy study, OCT = optical coherence tomography

## Introduction

Macular edema represents one of the main reasons that lead to decreased visual acuity in patients diagnosed with diabetic retinopathy. Macular edema is defined as a retinal thickening by pathological accumulation of extracellular fluid in the macular area.

Histologically, edema fluid is present in the outer plexiform layer and the internal nuclear [**7**].

Edema incidence increases with the duration and severity of diabetes and is more common in type II diabetes [**4**,**5**]. 

In WESDR, the incidence of macular edema after 10 years of development is of 20% in patients with diabetes mellitus type I, 25.4% in patients with type II diabetes insulin resistant and 13.9% of the patients with diabetes mellitus type II without insulin requirements.

This study showed that the incidence of macular edema also increased with the severity of retinopathy in both type I diabetes and in type II [**7**].

Diabetic macular edema is often associated with hard exudates, intraretinal hemorrhages and microaneurysms, which usually represent the extracellular fluid source. The long lasting intraretinal fluid can accumulate in the parafoveal cystic spaces forming cystoid macular edema [**6**].

The ETDRS defined the clinically significant macular edema as one of the following conditions (grading in order of severity):

1: retinal edema localized at less than 500 micrometers from the center of the macula;

2: hard exudates at less than 500 micrometers from the center of the macula, if they are associated with adjacent macular thickening;

3: at least one papillary diameter retinal thickening, located at less than a papillary diameter from the center of the macula [**7**].

The clinical diagnosis of macular edema involves biomicroscopic fundus examination by using either Goldman lens or lens Volk and stereoscopic fundus photography. These clinical diagnostic methods are subjective in assessing the present and the extent of the edema.

The last decade has brought significant achievements in terms of ocular imaging, allowing the objectification of retinal edema.

The optical coherence tomography (Ocular Coherence Tomography OCT) seems to be the most sensitive and reliable method for measuring the thickness of the edematous retina. It also shows the internal structure of the retina, the presence of intraretinal cystic spaces, and the presence of a thickened hialoide or vitreoretinal traction syndrome.

Regarding the therapeutic possibilities in well-selected cases, clinically significant macular edema treatment involves intravitreal injection of corticosteroids and antiangiogenic factors.

**Glucocorticoids** are used to treat diabetic macular edema for their anti-inflammatory effect, which can antagonize at least a part of the pathological processes involved in the occurrence of edema [**1**]. Glucocorticoids stabilize the capillary wall with consequent improvement of endothelial barrier function [**1**].

This process primarily occurs through the modulation of the synthesis and expression in endothelial cells of capillaries of molecules belonging to the class of intercellular tight junctions (occludin proteins) [**2**]. Secondly, the attraction of glucocorticoids inhibits leukocyte adhesion to the endothelium, and inhibits the further local inflammation [**2**]. On the other hand, the anti-inflammatory effect is due to the local inhibition of the synthesis of VEGF (vascular endothelial growth factor), known as the factor that increases the capillary permeability [**2**]. Secondly, glucocorticoids inhibit leukocyte adhesion to the endothelium, inhibiting the further local inflammation. On the other hand, the anti-inflammatory effect is due to the local inhibition of VEGF (vascular endothelial growth factor) synthesis also known as the factor that increases the capillary permeability.

**Glucocorticoid triamcinolone** acetonide seems to be most appropriate in this aspect. It is presented in vials as a white crystalline suspension, 40 mg triamcinolone in 1 ml solution (e.g. Vitreal S, very frequently used).

VEGF (vascular endothelial growth factor) was first discovered as a factor that increases vascular permeability and even called “vascular permeability factor “. The secretion of VEGF in the retina can cause increased capillary permeability and barrier damage. For this reason, it is considered that VEGF plays an important role in diabetic macular edema. Furthermore, the concentration of vitreal VEGF correlates with the severity of the macular edema [**3**].

**AntiVEGF agents** are representations of pegaptanib sodium, bevacizumab and ranibizumab, listed in a chronological order of their occurrence. Bevacizumab is the most commonly used antiVEGF agent [**3**].

**Bevacizumab** (e.g. Avastin, Genentech) is an anti-VEGF monoclonal antibody approved by the FDA in February 2004 for intravenous metastatic colorectal cancer. Intravitreal injection is used off-label and bevacizumab seems as effective as ranibizumab, but is considerably less expensive.

## Material and methods

The present study followed up for a period of at least six months patients diagnosed with clinically significant macular edema and treated with intravitreal injection of triamcinolone acetonide or triamcinolone acetonide in combination with bevacizumab .

The patients’ evolution was followed in terms of possible post intervention complications, functional parameters (visual acuity) and OCT parameters of macular region (mean retinal thickness in the center of the fovea - MCRT, total macular volume - TMV).

Thus, based on selectivity 295 patients (460 eyes) were selected and divided into 3 groups as it follows:

- Group A: 90 patients (161 eyes) treated with intravitreal injection of triamcinolone acetonide;

- Group B: 112 patients (180 eyes) treated with intravitreal injection of triamcinolone acetonide and bevacizumab;

- Group C: 93 patients (119 eyes), representing the control group.

Patients were randomly assigned for each group.

The assessment of changes in visual acuity is expressed by won or lost lines of visual acuity.

The data considered are the visual acuity measured from the first injection and at one month, three months and at least six months from the first intervention.

## Results

The group description depending on the stage of diabetic retinopathy and visual function at baseline showed a visual acuity of less than 0.3 in each of the three groups, **Table 1**.

**Table 1:** The initial visual acuity, acording to the diabetic retinopathy stage

	Stage	Initial VA	Initial VA	Initial VA	Initial VA
		≤0,1	>0,1 - ≤0,2	>0,2 - ≤0,3	>0,3
group A	Moderate NDR	15 (9,31%)	8 (4,96%)	7 (4,34%)	15 (9,31%)
	Severe NDR	32 (19,87%)	10 (6,21%)	6 (3,72%)	7 (4,34%)
	PDR	41 (25,46%)	11 (6,83%)	5 (3,10%)	4 (2,48%)
Group B	Moderate NDR	19 (10,55%)	5 (2,77%)	4 (2,22%)	15 (8,33%)
	Severe NDR	25 (13,88%)	9 (5%)	4 (2,22%)	11 (6,11%)
	PDR	68 (37,77%)	18 (10%)	0	2 (1,11%)
Control group	Moderate NDR	11 (9,24%)	3 (2,52%)	6 (5,04%)	16 (13,44%)
	Severe NDR	17 (14,28%)	5 (4,20%)	4 (3,36%)	5 (4,20%)
	PDR	33 (27,73%)	13 (10,92%)	5 (4,20%)	1 (0,84%)

In group A, who underwent intravitreal triamcinolone acetonide injection, the results showed that 38% of the patients had visual function improvements, 34% of them had unchanged visual function and in 28% patients, a poor outcome was registered. Percentages have slightly changed at three months and at least six months after the first intravitreal injection, **Table 2**.

**Table 2:** Group A, visual acuity outcome

	VA improvement	Same VA	VA drop
At 1 month	61 (37,88%)	55 (34,16%)	45 (27,95%)
At 3 months	65 (40,37%)	52 (32,29%)	44 (27,32%)
At 6 months	62 (38,50%)	51 (31,67%)	48 (29,81%)

**Table 3** presents the evolution of visual acuity in group A according to the number of lines gained or lost at the eye chart.

**Table 3:** Group A, acuity lines gained or lost (compared with VA at startup)

	Acuity lines	≤- 3	-2	-1	0	+1	+2	≥3
At 1 month	eyes	11	14	20	55	23	17	21
	(eye %)	6,83%	8,69%	12,42%	34,16%	14,28%	10,55%	13,04%
At 3 months	eyes	7	13	24	52	34	20	11
	(eye %)	4,34%	8,07%	14,90%	32,29%	21,11%	12,42%	6,83%
At 6 months	eyes	8	12	28	51	33	15	14
	(eye %)	4,96%	7,45%	17,39%	31,67%	20,49%	9,31%	8,69%

Thus, at 1 month, 13% of the eyes showed at least 3 lines improvement in visual acuity. This percentage decreased at 6 months to about 9%. 

The percentage of eyes which showed a decrease with at least 3 lines fell from about 7% to 5% at 6 months after the first injection.

In **group B**, 53% of the patients had a visual function improvement at three months after the therapy initiation. This percentage fell to 48 at 6 months, **Table 4**.

**Table 4:** Group B, visual acuity outcome

	VA improvement	Same VA	VA drop
At 1 month	91 (50,55%)	61 (33,88%)	28 (15,55%)
At 3 months	96 (53,33%)	67 (37,22%)	17 (9,44%)
At 6 months	87 (48,33%)	65 (36,11%)	28 (15,55%)

The number of eyes that have lost at least 3 lines at eye chart remains small (around 3% at one month and over 3% at 6 months). Also, over 36% of the patients gained at least two lines on the eye chart in all three intervals, **Table 5**.

**Table 5:** Group B, acuity lines gained or lost (compared with VA at startup)

	Acuity lines	≤- 3	-2	-1	0	+1	+2	≥3
At 1 month	eyes	5	11	12	61	23	42	26
	(eye %)	2,7%	6,11%	6,66%	33,88%	12,77%	23,33%	14,44%
At 3 months	eyes	2	7	8	67	24	44	28
	(eye %)	1,1%	3,88%	4,44%	37,22%	13,33%	24,44%	15,55%
At 6 months	eyes	6	13	9	65	21	40	26
	(eye %)	3,3%	7,22%	5,00%	36,11%	11,66%	22,22%	14,44%

For the control group, most eyes maintained the same visual acuity at 6 months after the enrollment (48%). Almost a third had declines in visual acuity and only 20% showed an improvement in the visual function, **Table 6** and **Table 7**.

**Table 6:** Group C, visual acuity outcome

	VA improvement	Same VA	VA drop
At 3 months	29 (24,36%)	64 (53,78%)	26 (21,84%)
At 6 months	24 (20,16%)	57 (47,89%)	38(31,93%)

**Table 7:** Group C, acuity lines gained or lost (compared with VA at startup)

	Acuity lines	≤- 3	-2	-1	0	+1	+2	≥3
At 3 months	eyes	4	8	14	64	15	10	4
	(eye %)	3,36%	6,72%	11,76%	53,78%	12,60%	8,40%	3,36%
At 6 months	eyes	6	11	21	57	14	7	3
	(eye %)	5,04%	9,24%	17,64%	47,89%	11,76%	5,88%	2,52%

## Conclusions

The main purpose of the present study was to compare the functional and structural parameters in the two groups of patients. **Table 8** presents the visual acuity at 3 months and 6 months for the two groups, compared with the control group.

**Table 8:** Group A and B visual acuity outcome at 3 and 6 months, compared with the control group

	Group A 161 eyes	Group B 180 eyes	Group C 119 eyes	Group A - Group C	Group B - Group C	Group B vs Group A
	(TA)	(TA+BV)	(control group)	x	y	y-x
	(% eyes)	(% eyes)	(% eyes)	(% eyes)	(% eyes)	(% eyes)
VA improvement						
at 3 months	40,37%	53,33%	24,36%	16,01%	28,97%	12,96%
at 6 months	38,50%	48,33%	20,16%	18,34%	28,17%	9,83%
Same VA						
at 3 months	32,29%	37,22%	53,78%	-21,49%	-16,56%	4,93%
at 6 months	31,67%	36,11%	47,89%	-16,22%	-11,78%	4,44%
VA drop						
at 3 months	27,32%	9,44%	21,84%	5,48%	-12,40%	-17,88%
at 6 months	29,81%	15,55%	31,93%	-2,12%	-16,38%	-14,26%

It could be seen that a significantly higher percentage of eyes had visual function improvements (28.17% Group B, 18.34% Group A, p statistically significant p < 0.05) in group B (Triamcinolone + Bevacizumab).

A significantly lower percentage of eyes with decreased visual acuity compared to group A (17.88% less at 3 months and 14.26% less in 6 months, p statistically significant, p < 0.05) was recorded in group B.

Thus, a combined administration of triamcinolone acetonide and bevacizumab is more effective regarding the visual function outcome for short and medium term.

The results showed a visual acuity improvement at 1 month and 3 months after the triamcinolone injection, differences between the average visual acuity at 1 month and 3 months was statistically significant compared to the initial visual average. Visual acuity at 6 months is greater than the baseline, but this difference is not statistically significant. 

This means that triamcinolone effectiveness fell at 6 months after the administration.

Patients who received bevacizumab and triamcinolone showed an increase in the visual acuity at one month and three months after the injection. Visual acuity dropped six months later for these patients. These visual changes have the same trend as the triamcinolone group, except for the difference between the average visual acuity obtained at 6 months where the visual acuity is higher than the baseline average. These values are statistically significant (p < 0.05).

For group B (intravitreal injection of triamcinolone and bevacizumab), a significantly higher number of patients had increases in the visual acuity compared with group A, the average visual acuity being maintained at 6 months after the treatment initiation (p statistically significant, p < 0.05).

The combined administration of the angiogenesis inhibitor and glucocorticoid is superior in terms of visual function on short and medium term, compared to the administration of triamcinolone.
